# Biology of *Bedellia somnulentella* (Lepidoptera: Bedelliidae) Associated with Wild *Ipomoea* spp. (Convolvulaceae) as Host Plants

**DOI:** 10.3390/insects17040425

**Published:** 2026-04-16

**Authors:** Maria Jéssica dos Santos Cabral, Rodrigo Almeida Pinheiro, Isabel Moreira da Silva, José Barbosa dos Santos, Muhammad Haseeb, Marcus Alvarenga Soares

**Affiliations:** 1 Center for Biological Control, College of Agriculture and Food Sciences, Florida A&M University, Tallahassee, FL 32307, USA; jessica1.cabral@famu.edu (M.J.d.S.C.); muhammad.haseeb@famu.edu (M.H.); 2 Departamento de Agronomia, Universidade Federal dos Vales do Jequitinhonha e Mucuri, Diamantina 39100000, MG, Brazil; almeida.rodrigo@ufvjm.edu.br (R.A.P.); ibelmoreira@yahoo.com.br (I.M.d.S.); jbarbosa@ufvjm.edu.br (J.B.d.S.)

**Keywords:** biological cycle, defoliating pest, microlepidoptera, sweet potato

## Abstract

*Bedellia somnulentella* (Lepidoptera: Bedelliidae) is an invasive, leaf-mining, and defoliating pest of sweet potatoes (*Ipomoea batatas* L.) that has recently established in Brazil. Its colonization and infestation levels in cultivated fields are influenced by the availability of wild *Ipomoea* species that sustain populations during off-season periods. The objective was to evaluate the biology and life history of *B. somnulentella* feeding on wild plants of the genus *Ipomoea* and on *I. batatas* cv. Beauregard. Vegetative and reproductive parts of *Ipomoea* plants were collected and cultivated, and the biology and life history of *B. somnulentella* were studied using twenty adult pairs of the insect per host plant in a climate-controlled room. The wild species *Ipomoea hederifolia* L., *Ipomoea indica* (Burm.f.) Merr., *Ipomoea purpurea* L., and cultivated *I. batatas* were used for the assays. The experiment followed a completely randomized design with ten replicates. Hatching, larval stages, prepupa, pupa, and adult phases were observed and recorded daily. Variations in the coloration of *B. somnulentella* larvae feeding on leaves of *I. hederifolia*, *I. indica*, and *I. purpurea* were observed. The survival and development of *B. somnulentella* were higher on *I. batatas* and *I. hederifolia* than on *I. indica* and *I. purpurea*, mainly during the larval and adult stages. The results provide information on infestation in alternative wild hosts and on biological aspects of *B. somnulentella*.

## Simple Summary

The larvae of *Bedellia somnulentella* (Lepidoptera: Bedelliidae) form mines in young and mature leaves, completely consuming the leaf mesophyll and leaving a transparent epidermis, causing leaf wilting, reduced photosynthesis, and death of the host plants. This insect has expanded into new regions, becoming a growing concern for farmers; however, its development on host plants is still poorly understood. In this study, we investigated the life cycle of *B. somnulentella* and its survival on wild *Ipomoea* spp. and cultivated *I. batatas* plants. By understanding how its population grows, farmers and technicians can improve monitoring and develop more effective and environmentally friendly management strategies, helping to reduce losses and protect food production.

## 1. Introduction

Herbivores have evolved adaptations to their host plants across diverse environments [1]. Most herbivorous insects exhibit selective feeding behavior, particularly under free-choice conditions [2]. Nevertheless, spatial and temporal variability in the availability of suitable hosts is common across ecosystems [2]. Insect–host plant interactions are co-evolutionary, with host plants influencing feeding, mating, oviposition behavior, and also insect development and population growth [3,4,5], with higher rates observed on hosts that are well adapted [6] than on hosts that are nutritionally inadequate or possess stronger defense mechanisms [7,8,9].

The microlepidopteran *Bedellia somnulentella* (Zeller, 1847) (Lepidoptera: Bedelliidae), an invasive defoliator of sweet potato *Ipomoea batatas* (L.) (Convolvulaceae) plants [10], originating in Eurasia, has spread throughout Africa, the Americas, and Oceania. In South America, this insect was initially recorded in Peru in 1914, with a specimen collected and preserved in the Natural History Museum, London (1914), and in Brazil in 2018, damaging the leaves of *I. batatas* in Diamantina, state of Minas Gerais [11]. *Bedellia somnulentella* is a polyphagous pest, but its larvae feed most frequently on plants of the Convolvulaceae family [12,13,14,15]. Larval leaf-mining activity, with the selective consumption of mesophyll tissues while leaving the epidermis intact, induces structural and functional disruption of the leaf, with effects on photosynthetic performance and overall host plant integrity [16].

In agricultural landscapes, several wild *Ipomoea* species (e.g., *Ipomoea hederifolia* L., *Ipomoea indica* (Burm.f.) Merr., *Ipomoea purpurea* L.) are prevalent weeds that often coexist with *I. batatas* cultivation [12]. The ability of *B. somnulentella* to utilize this range of host plants, including these wild *Ipomoea* species, is an important factor in its persistence and success as an invasive species [12]. These wild plants can serve as refuges during the off-season of the primary host and as reservoirs that maintain and accelerate the pest’s population growth, facilitating its establishment in new areas, subsequent dispersal, and pressure on crops.

Despite the documented presence of *B. somnulentella* on wild *Ipomoea* species [12], a comprehensive understanding of the comparative biological performance (e.g., ability to complete the cycle, developmental duration, survival, fecundity) and feeding efficiency of the pest in these hosts has remained unexplored. Previous studies have primarily focused on documenting new host associations or general descriptions of life stages [11,12]. Therefore, this study addresses a knowledge gap by quantitatively assessing how different *Ipomoea* species affect the life history traits and leaf consumption of *B. somnulentella.* By understanding how its population grows, farmers and technicians can improve monitoring and develop more effective and environmentally friendly management strategies, helping reduce losses and protect food production [10].

Oviposition and the survival of insect immature stages have been shown to vary among host species [2,3,4,17]. Whereas specialist insects prefer high-quality host plants, polyphagous species are typically less selective [5]. Thus, the life history of *B. somnulentella* and its survival during *I. batatas* off-season periods on alternative host plants require further investigation. This study aimed to evaluate the life history of *B. somnulentella* feeding on wild *Ipomoea* spp. and cultivated *I. batatas*.

## 2. Materials and Methods

### 2.1. Experiment Locations

The experiments were conducted in a greenhouse at the Crop Sector and the Entomology Laboratory of the Universidade Federal dos Vales do Jequitinhonha e Mucuri (UFVJM, campus JK) in Diamantina, Minas Gerais state, Brazil. The host plant leaves used in the experiments were all obtained from greenhouse-grown plants.

### 2.2. Rearing of Bedellia somnulentella

Pupae of *B. somnulentella* were collected from leaves of *I. batatas* cv. Beauregard and kept in Petri dishes (49 × 13 mm) under laboratory-controlled environmental conditions (25 ± 2 °C, 70 ± 10% relative humidity, and a 12:12 h light–dark photoperiod). The pupae were kept in the laboratory in Petri dishes until adult emergence. These adults were placed in wooden cages (0.35 × 0.35 × 0.30 m) covered with organza fabric and with a glass front opening, kept at room temperature, with branches of *I. batatas* fixed to the glass in a plastic tray (7.5 cm high, 22 cm wide, and 30 cm long) containing water and closed with flexible polyurethane foam. New branches of *I. batatas* and water were added when necessary to maintain the rearing of this insect. A sugar solution (5.0%, *w*/*v*) was placed inside the cage twice a week as food for *B. somnulentella* adults.

### 2.3. Plants Tested

*Ipomoea alba* L., *Ipomoea cairica* L., *Ipomoea hederifolia* L., *Ipomoea indica* (Burm.f.) Merr., *Ipomoea maurandioides* Meisn. and *Ipomoea purpurea* L. were collected and identified in Diamantina, state of Minas Gerais, Brazil [12]. Based on preliminary observations, *I. hederifolia*, *I. indica*, and *I. purpurea* were selected for the bioassays due to their capacity to support the development of *B. somnulentella* adequately. Tested plants were grown in pesticide-free environments in pots with a capacity of 1 L (insect biology assessment) and 10 L (leaf area assessment) in a greenhouse and were irrigated daily by sprinkler irrigation. Three branches were transplanted per pot.

### 2.4. Bioassay I: Developmental and Reproductive Biology of B. somnulentella on Ipomoea Host Plants

The biological parameters of *B. somnulentella* were assessed under a no-choice experimental design to evaluate its development on *Ipomoea batatas* cv. Beauregard, *I. hederifolia*, *I. indica*, and *I. purpurea*. Briefly, 1 L pots with each host plant were placed in wooden cages with 20 couples of *B. somnulentella* adults (≤24 h old) per cage, and the cages were observed in a climate-controlled room at 25 ± 2 °C, 70 ± 10% relative humidity, and a 12 h photoperiod. Oviposition and hatching of this insect were observed and recorded daily. Newly hatched larvae were transferred with a brush and tweezers to Petri dishes (15 cm in diameter, 1.5 cm in height) with fresh leaves of *I. batatas*, *I. indica*, *I. hederifolia*, or *I. purpurea*. The petioles of these leaves were inserted into glass test tubes with cotton soaked in water to reduce water loss. Larval mortality was recorded, and the leaves were replaced when necessary. Newly formed pupae were transferred to Petri dishes with cotton balls soaked in water to reduce moisture loss, covered with gauze, and observed daily until adults emerged. Duration and survival of the egg, larva, prepupal, and pupal stages and adult longevity were obtained. Females and males emerged within 24 h per host plant, were mated, and were kept in wooden cages (13 cm in diameter, 17 cm in height) for oviposition, and the number of eggs was counted every 24 h until the death of the adults. Changes were observed in 40 larvae, 10 per host plant. Viability, duration, and changes in morphological characteristics in all stages were evaluated daily until adults emerged. Adult longevity was assessed in 40 individuals (10 per host plant) of *B. somnulentella*, isolated in 0.5 L plastic containers and covered with plastic film with ventilation holes (8–10 mm in diameter).

### 2.5. Bioassay II: Leaf Area Consumption

Leaf area consumption by *B. somnulentella* was evaluated in a no-choice experiment. For this, five leaves from each host plant, isolated in cages, were removed after 30 days of infestation by adult *B. somnulentella* and photographed with a Poco F3 camera (Xiaomi, Beijing, China) at 1080 × 2400 pixels. The leaf area of *I. batatas*, *I. hederifolia*, *I. indica*, and *I. purpurea*, consumed by *B. somnulentella*, was evaluated with LeaFImage Software version 3—leaf area meter (UFVJM) by reading the pixels and separating those with previously determined colors, that is, the green area of the leaves [10]. A monochromatic color (red) was used to highlight the leaf area consumed by *B. somnulentella* by replacing the target color. The number of pixels forming the image was calculated by summing the total area of the leaves and the number of replaced pixels, allowing the determination of the area consumed by *B. somnullentela*, with a scale defined in cm^2^ for its calculation [10].

### 2.6. Statistical Analysis

The normality of the distribution of residuals and the homogeneity of variances were assessed using the Shapiro–Wilk and Levene tests, respectively. Analysis of variance (ANOVA) was applied after verifying these assumptions. When it resulted in a significant *p*-value by the F test (*p* ≤ 0.05), the means were compared by the Tukey test (*p* ≤ 0.05). The Dunn test verified the Kruskal–Wallis test and the differences between the groups when the parametric tests’ assumptions were not met. Statistical analysis was performed using the R program version 4.3.2 [18,19].

## 3. Results

### 3.1. The Developmental and Reproductive Biology of B. somnulentella on Ipomoea Host Plants

The duration of the development period of *B. somnulentella* from egg deposition to adult emergence, as well as the duration of each developmental stage (egg, larva, prepupa, pupa, and adult), varied among the *Ipomoea* species tested (*I. batatas*, *I. hederifolia*, *I. indica*, and *I. purpurea*) (Table 1). Statistical analyses revealed significant differences in the total development period (F = 9.75, *p*= 0.02), egg stage (F = 14.11, *p* = 3.08), pupal stage (F = 8.22, *p* = 0.00), and adult longevity (F = 7.31, *p* = 0.00). No significant differences were observed in the prepupal stage (F = 1, *p* = 0.40). Detailed durations for each stage are presented in Table 1.

**Figure 1:**
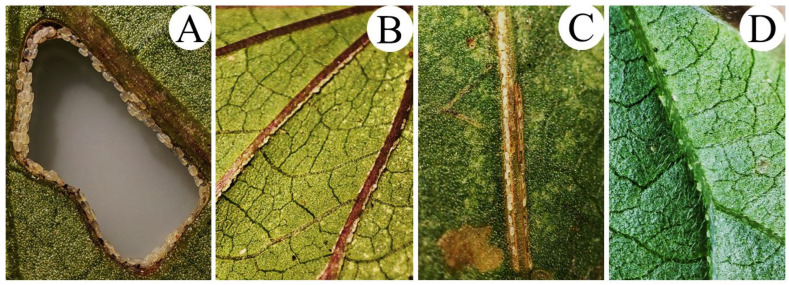
Eggs of *Bedellia somnulentella* (Lepidoptera: Bedelliidae) on leaves of *Ipomoea batatas* (**A**), *Ipomoea indica* (**B**), *Ipomoea hederifolia* (**C**), and *Ipomoea purpurea* (**D**).

*Bedellia somnulentella* eggs are translucent white with black dots on the upper edges near the larvae’s emergence point (Figure 1). These eggs are hemispherical and are usually laid on the lower (abaxial) surface attached to the central and secondary veins of the host plant leaves (Figure 1).

**Figure 2:**
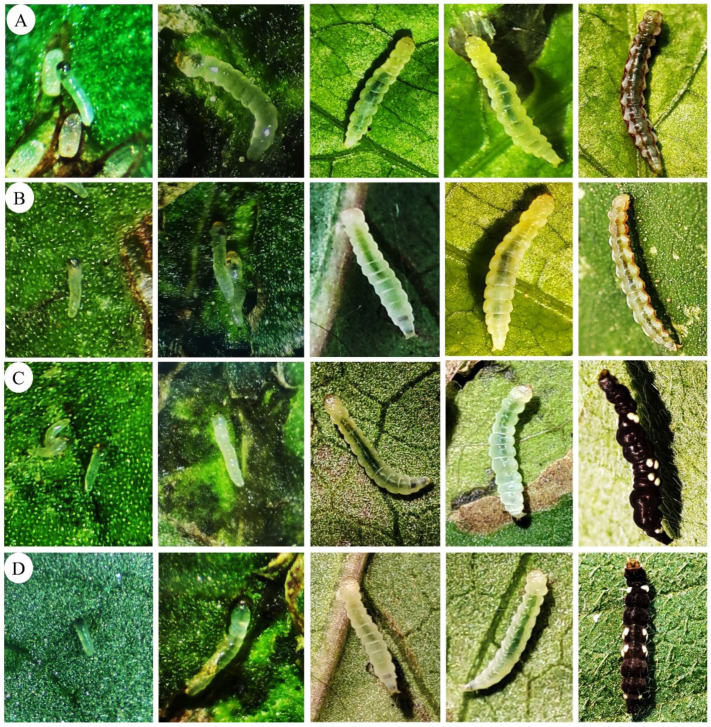
Larvae of different instars of *Bedellia somnulentella* (Lepidoptera: Bedelliidae) feeding on leaves of *Ipomoea batatas* (**A**), *Ipomoea hederifolia* (**B**), *Ipomoea indica* (**C**), and *Ipomoea purpurea* (**D**) (Convolvulaceae).

*Bedellia somnulentella* larvae went through five instars on *I. batatas* (A), *I. hederifolia* (B), *I. indica* (C), and *I. purpurea* (D) (Figure 2). The morphological characteristics of *B. somnulentella* larvae varied between instars and host plants (Figure 2).

Morphological characteristics of *B. somnulentella* larvae fed on *I. indica* and *I. purpurea* differed according to their instars (Figure 2). First-instar larvae fed on *I. indica* and *I. purpurea* are translucent with a visible light brown head capsule and greatly reduced or inconspicuous thoracic legs; second-instar larvae are light green with a black head capsule; third-instar larvae are white with a dark green color; fourth-instar larvae are dark green throughout their body; and fifth-instar larvae are black with seven light yellow spots on each side, totaling fourteen light yellow spots throughout their body. In later instars (e.g., third to fifth), both thoracic legs and prolegs become more evident, as can be observed in Figure 2.

**Figure 3:**
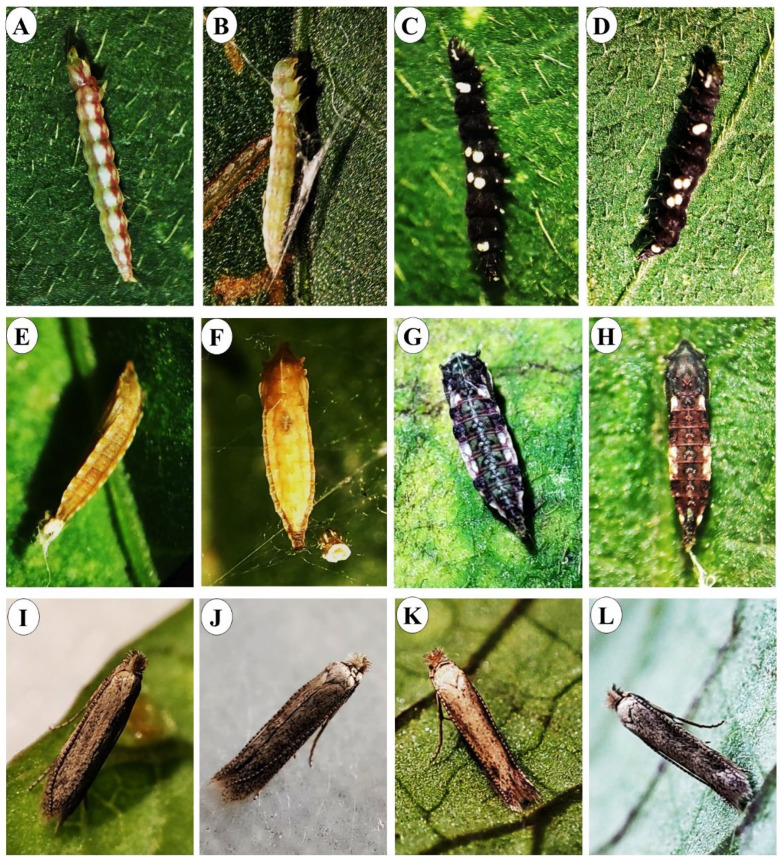
Prepupa, pupa, and adults of *Bedellia somnulentella* (Lepidoptera: Bedelliidae) in *Ipomoea batatas* (**A**,**E**,**I**), *Ipomoea hederifolia* (**B**,**F**,**J**), *Ipomoea indica* (**C**,**G**,**K**), and *Ipomoea purpurea* (**D**,**H**,**L**).

Prepupae and newly formed pupae of *B. somnulentella* are reddish green, changing to light and dark brown as they approach adult emergence in *I. batatas* and *I. hederifolia* (Figure 3A,B,E,F). In *I. indica* and *I. purpurea*, pupae are black with yellow spots on their sides and attached with silk threads under the leaves (Figure 3C,D) or dark black with white spots on the sides and dark brown above (Figure 3G,H). Adults of *B. somnulentella* arising from pupae in host plants are bronze-yellow moths 4.0 mm long with fringed hindwings (Figure 3I,J). At rest, the forewings cover the hindwings due to the insect’s habit of landing on the abaxial end of the leaf (Figure 3K,L).

**Figure 4:**
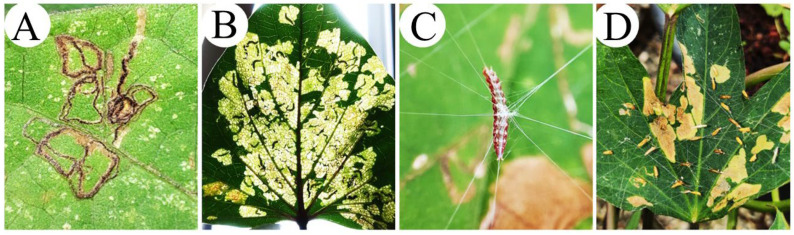
*Bedellia somnulentella* (Lepidoptera: Bedelliidae) mines on *Ipomoea* spp. (Convolvulaceae) leaves in a zigzag pattern (**A**), larvae on larger mines (**B**), larvae on silk threads (**C**), and pupae attached to silk threads (**D**).

The feeding behavior of *B. somnulentella* larvae varied among instars, with early instars making zigzag mines and later instars making larger, rounded mines on young and mature leaves of host plants (Figure 4A,B). These larvae produced silk threads on leaf surfaces to move between plants and remained attached during the prepupal and pupal stages without forming a cocoon (Figure 4C,D).

The survival and development of *B. somnulentella* varied among the evaluated *Ipomoea* host species throughout all developmental stages (Table 2). During the egg stage, the mean viability of *B. somnulentella* eggs was highest on *I. batatas*, differing from those on other species. In the larval stage, survival was again highest on *I. batatas* and *I. hederifolia*, which differed from *I. indica* and *I. purpurea*, where larvae exhibited slower growth and reduced feeding activity. In the prepupal and pupal stages, *I. batatas* maintained conditions for the highest survival rates of *B. somnulentella*, followed by *I. hederifolia*, while the prepupae and pupae developed on *I. indica* and *I. purpurea* showed the lowest survival, with several individuals failing to pupate properly. Adult emergence was significantly greater in specimens grown on *I. batatas*, compared to *I. hederifolia*, *I. indica*, and *I. purpurea* (Table 2). Adults from *I. batatas* were larger, more active, and morphologically complete, whereas those from *I. indica* and *I. purpurea* were fewer and often deformed. Overall, *B. somnulentella* showed the highest survival and successful development on *I. batatas* and *I. hederifolia*, indicating these species as the most suitable hosts for population maintenance, while *I. indica* and *I. purpurea* were less favorable. Statistical analysis (two-way ANOVA, *p* < 0.001 for species and stage; Tukey’s test, *p* < 0.05) confirmed that host plant species affected the survival of *B. somnulentella* at all developmental stages, with minimal survival observed on *I. purpurea* and optimal biological performance on *I. batatas* (Table 2).

### 3.2. Leaf Area Consumption

**Figure 5:**
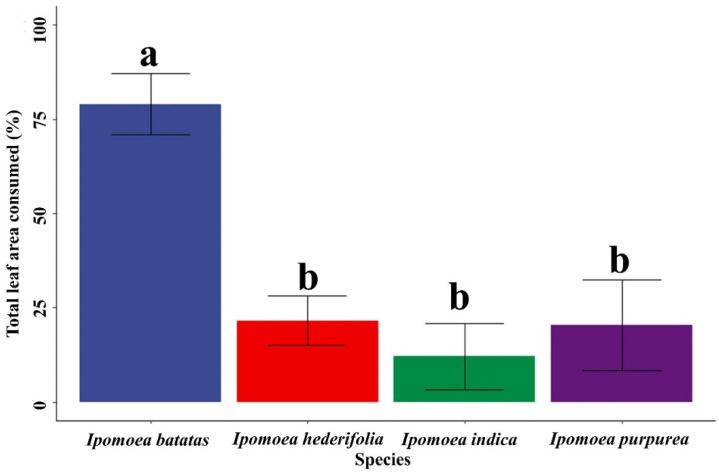
Leaf area (means—bars and standard deviation—vertical lines) of *Ipomoea batatas*, *Ipomoea indica*, *Ipomoea hederifolia*, and *Ipomoea purpurea* consumed by *Bedellia somnulentella* (Lepidoptera: Bedelliidae). Means followed by the same letter do not differ by Tukey’s test at a significance level of 5%.

**Figure 6:**
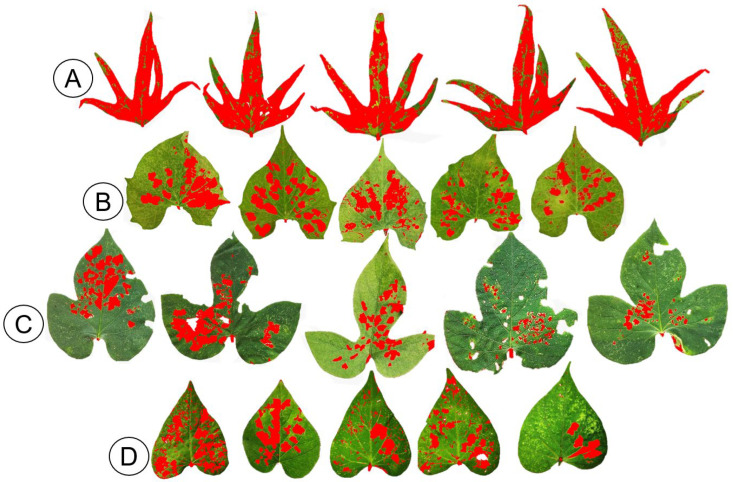
Representations of the leaf area of *Ipomoea batatas* (**A**), *Ipomoea indica* (**B**), *Ipomoea hederifolia* (**C**), and *Ipomoea purpurea* (**D**) consumed by *Bedellia somnulentella* (Lepidoptera: Bedelliidae) to assess damage severity. The red areas indicate the leaf tissue consumed by the insect.

The leaf area consumed by *B. somnulentella* varied among the host plants, ranging from 78.98% to 12.78% (Gl = 3, F = 89.11, *p* = 3.36) (Figure 5). Specifically, the percentages of consumed leaf area were 78.98% for *I. batatas*, 21.86% for *I. hederifolia*, 12.78% for *I. indica*, and 20.17% for *I. purpurea*. Figure 6 provides visual representations of the damage severity on leaves of each *Ipomoea* species, illustrating how the consumed area was quantified by highlighting the affected regions.

## 4. Discussion

The similar durations of the egg and prepupal stages of *B. somnulentella* across *Ipomoea* species may be explained by specific factors, such as temperature and humidity. The duration of the egg stage is typically governed by embryonic development, which depends primarily on environmental factors such as temperature and humidity, rather than on the host plant [20]. Since the egg is deposited externally on the host plant but does not feed or metabolize plant compounds, the influence of *Ipomoea* species is minimal. The prepupal stage represents a physiological transition from feeding larva to pupa and involves internal reorganization [16]. Like the egg stage, it is primarily influenced by internal hormonal controls (ecdysteroids and juvenile hormones) and external environmental stimuli (temperature, photoperiod), rather than by the host plant species [21]. Although *Ipomoea* species can strongly affect larval stages, such as feeding due to differences in nutritional quality or chemical defenses, they have less impact on non-feeding stages, such as the egg and prepupa. The similar durations are likely due to these stages being developmentally fixed and less plastic in response to host plant variation. In contrast, the larval period is more sensitive to host plant quality.

The longer duration of the larval and pupal stages in *I. indica* and *I. purpurea* may be related to toxic or repellent compounds, such as alkaloids, tannins, and phenols, inhibiting the growth, feeding, or reproduction of insects and, therefore, may affect the development of pests such as *B. somnulentella* on plants of the *Ipomoea* genus [22]. Nutritional availability and quality may be other important factors, as the nutritional composition of leaves and edible parts may differ among different *Ipomoea* species [23]. More resistant plants or those with less favorable traits can reduce the insect life cycle and survival [24,25,26]. However, the ability of *B. somnulentella* to complete its life cycle on wild *Ipomoea* species suggests that these plants function as reservoirs during off-season periods, enabling persistence in the landscape and facilitating reinfestation of *I. batatas* crops. This ecological role of alternative hosts has important implications for pest management, as weed control and habitat management may reduce population carryover between cropping cycles.

The increased longevity of *B. somnulentella* by three and four days on *I. indica* and *I. purpurea*, respectively, highlights variation in this parameter, consistent with previous reports for *Phyllocnistis citrella* Stainton (Lepidoptera: Gracillariidae), whose larval and pupal longevity varied by up to four days on *Citrus paradisi* and 6.5 days on *C. sinensis* [27]. The development period of *P. citrella* was shorter in *C. sinensis* than in *C. aurantifolia*, *C. limetta* Risso, *C. aurantium* Linnaeus, *C. paradisi*, and *C. reticulata* during two samplings of *Citrus* orchards than in *C. paradisi* and *C. limetta* [27]. The average period of the egg, larval, and pupal stages of *P. citrella* was 3.65, 8.95, and 7.5 days, respectively, in the laboratory on *C. sinensis* seedlings [28], with a total duration of the immature stages of 20.1 days. The development from egg to adult of *B. somnulentella* was about four weeks on *I. batatas* and *I. hederifolia*, with up to 12 generations per year and about 50–60 eggs per female. Female insects typically oviposit on the most suitable available host plant, as it generally constitutes the sole food source for their larvae [29,30]. Host selection and acceptance are primarily influenced by semiochemicals and apparent plant traits [31].

The translucent white color with black dots on the upper ends near the emergence of larvae from *B. somnulentella* eggs is related to embryonic development, and the black dot on the upper end indicates the formation of the initial chitinous pigmentation of the embryo’s head [32]. The observed variations in larval coloration and morphology of *B. somnulentella* across different host plants, as detailed in Section 3, highlight significant phenotypic plasticity. For instance, fifth instar larvae on *I. indica* and *I. purpurea* were black, while those on *I. batatas* and *I. hederifolia* showed distinct coloration patterns in later instars, such as pink spots and lateral stripes. This morphological divergence, particularly the differences in coloration, is common in Lepidoptera and can be influenced by host plant chemistry and nutritional quality [33]. Variation in the color of larvae on different host plants was reported for *Drymoea veliterna* (Druce) (Lepidoptera: Geometridae) on trees of the genus *Croton* L. (Euphorbiaceae) in Colombia [33]. Knowing the color variations of the species across different hosts is important to avoid misidentification. Such variations may also reflect adaptive responses to different host plant environments, potentially impacting crypsis or signaling to predators. The five instars of *B. somnulentella* feeding on *I. batatas* are similar to those reported for this pest in California and southwestern Virginia, USA; Diamantina, Minas Gerais state, Brazil [16,34]; and Romania [35]. Five is the most common number of larval instars in Lepidoptera species [36].

Variations in the feeding behavior of *B. somnulentella* larvae are evident across developmental stages. Early instars typically construct narrow zigzag mines, whereas from the third instar onward, they produce larger mines on both young and mature leaves. This pattern is consistent with the feeding behavior observed in many leaf-mining larvae [37]. Mining functions as a strategy that enhances feeding efficiency while reducing exposure to predators, thereby increasing survival during the earliest stages of development [38]. As larvae grow, their nutritional demands increase, leading them to construct progressively larger mines to access more leaf tissue [39]. Increased nutrient intake, even in the presence of plant defenses, supports larval growth and development in later instars [39].

The production of silk threads by *B. somnulentella* may be related to larval movement and protection against predators [10,39], and its pupae hanging on threads above the leaf surface may protect them against predators. Similar behavior was observed in larvae of *Thyrinteina arnobia* (Stoll) (Lepidoptera: Geometridae) hanging on silk threads and avoiding predation by *Podisus fuscescens* (Dallas) (Hemiptera: Pentatomidae) [39], referred to as *P. distinctus*.

The lower mortality of larvae, pupae, and adults of *B. somnulentella* in *I. batatas* and in the prepupal phase, similar between hosts, and the higher mortality of adults in *I. indica* and *I. purpurea* are probably due to the nutritional composition of the plant, such as vitamins and minerals, carbohydrates, starch and fiber, vitamin A, vitamin C, and B vitamins (B1, B2, B3, and B6) and minerals such as potassium, manganese, and calcium, in addition to proteins, antioxidants, natural sugars, and fats [40]. The lower larval survival rate of *B. somnulentella* observed on *I. indica* and *I. purpurea*, compared to *I. batatas*, may also be partially attributed to the greater sensitivity of the early larval instars to the host plant’s defenses. Young larvae, with their less developed mouthparts and immature detoxification systems, are often more vulnerable to toxic secondary compounds (e.g., alkaloids, tannins, and phenols) and physical barriers (e.g., trichomes, leaf toughness) present in wild plants [21,24]. Mortality in the early instars contributed to the lower overall larval survival recorded on these wild hosts. This initial vulnerability may exert strong selective pressure, favoring survival on more compatible hosts or the evolution of detoxification mechanisms in populations that persist on plants with more defenses. Understanding this differential sensitivity is important for elucidating mechanisms underlying host specificity and for developing management strategies that target the pest’s most vulnerable stages.

Variations in leaf consumption by *B. somnulentella* in *I. batatas*, *I. indica*, *I. hederifolia*, and *I. purpurea*, between 78.98 and 12.78%, are probably related to the morphological, physical, and chemical factors of the leaves of each plant, such as area, shape, nutrition, and anatomical modifications increasing or decreasing the interaction between plants and insects [41] and, consequently, preference for oviposition and feeding. *Phyllocnistis citrella* did not damage Sunki mandarin leaves, but 86% of Rangpur lime had signs of feeding by this pest [27]. Variation in host selection and damage by *P. citrella* has been attributed to morphological factors such as softness or hardness, presence or absence of trichomes, and leaf shape and color [42]. This variation in leaf consumption suggests that *B. somnulentella* exhibits selective feeding behavior influenced by specific leaf traits of different *Ipomoea* species. These findings highlight the roles of plant morphology and chemistry in mediating insect–plant interactions and may help identify host plants with greater resistance potential for pest management strategies.

The greater preference of *B. somnulentella* larvae for *I. batatas* species is probably related to the higher nutrient content of the Beauregard cultivar and anatomical modifications of their leaves [43]. As wild plants do not produce tuberous roots, they may present nutritional deficiencies, which can influence the population density of the pest. The oviposition site and offspring development vary with leaf characteristics [11]. This is important for lepidopterans with low-mobility larvae; therefore, it is dependent on the choice of feeding site made by adult females. The choice of females to lay eggs on hosts with high-quality food resources is particularly important for leaf miners, as many of them feed only at or near oviposition sites [44]. This result reinforces that the feeding and oviposition preferences of *B. somnulentella* are strongly influenced by the nutritional quality and morphological traits of the host plant, with better resources in the Beauregard cultivar. Such preferences are important for species survival, as female selection of optimal oviposition sites directly impacts larval development and overall pest population dynamics.

The results indicate that wild *Ipomoea* species, although less nutritionally favorable for *B. somnulentella* development than *I. batatas*, are important in the ecology of this pest as alternative hosts. The pest’s ability to complete its life cycle, even with lower reproductive success or a longer development time, is important for maintaining its populations in the environment, especially during periods of absence or low availability of the cultivated host. This persistence in wild hosts ensures the species’ survival and contributes to the expansion of its range and the infestation pressure on sweet potato crops. Understanding how these wild hosts influence population growth rates and pest dispersal can help to mitigate the impacts of *B. somnulentella* as an invasive species.

## 5. Conclusions

There were five larval instars of *B. somnulentella* on the host plants, with morphological differences in the larval, prepupal, and pupal stages. Larvae of *B. somnulentella* on *I. batatas* and *I. hederifolia* are translucent in the early instars, with a light green color throughout their body and dark green in the digestive tract and pink spots on all thoracic and abdominal segments and four lateral stripes in the fourth and fifth instars. In *I. indica* and *I. purpurea*, the first instars are translucent, while the second, third, and fourth instars are dark green all over their bodies, and the fifth instars are black with seven light yellow spots on each side, totaling fourteen spots on their bodies.

Insect development varied among host plants, with individuals reared on *I. batatas* (Beauregard) exhibiting shorter development times and higher survival rates than those reared on weeds such as *I. hederifolia*, *I. indica*, and *I. purpurea*, which are likely less nutritionally suitable and may contain deterrent compounds. These differences suggest that host plant quality directly affects feeding and oviposition behavior, as well as the pest’s biological performance.

*Bedellia somnulentella* consumed more leaves from *I. batatas* (78.98%) than from the wild plants *I. hederifolia* (21.86%), *I. indica* (12.78%), and *I. purpurea* (20.17%). Cultivated varieties such as *I. batatas* are often selected for traits that increase palatability and nutritional content, which may unintentionally make them more attractive and suitable for *B. somnulentella*. Wild species may possess physical defenses or chemical deterrents that reduce leaf consumption. However, the ability of *B. somnulentella* to complete its life cycle on wild *Ipomoea* species indicates that these plants may act as reservoirs that sustain populations outside cultivated fields, favoring the persistence and spread of this invasive pest.

## Figures and Tables

**Table 1:** Duration (mean ± standard error) of egg, larva, prepupa, pupa, and adult stages of *Bedellia somnulentella* (Lepidoptera: Bedelliidae) on *Ipomoea batatas*, *Ipomoea indica*, *Ipomoea hederifolia*, and *Ipomoea purpurea*.

Host Plant	Duration Stages (Days)				
	Egg	Larval	Prepupa	Pupa	Adult
*I. batatas*	8.6 ± 0.48 a	10.9 ± 0.87 a	1 ± 0.60 a	6.2 ± 0.13 a	6.6 ± 0.67 a
*I. hederifolia*	8.7 ± 0.48 a	11.9 ± 0.87 a	1 ± 0.60 a	6.3 ± 0.15 a	4.7 ± 0.70 b
*I. indica*	9 ± 0.69 a	13.2 ± 0.78 b	1 ± 0.67 a	7.5 ± 0.17 b	4.2 ± 0.78 c
*I. purpurea*	9 ± 0.84 a	13.7 ± 0.48 b	1 ± 0.71 a	7.4 ± 0.16 b	3.3 ± 0.67 d

Different letters within the same column indicate statistically significant differences according to Tukey’s test (*p* < 0.05).

**Table 2:** Mean survival (%, mean ± standard error) of *Bedellia somnulentella* (Lepidoptera: Bedelliidae) on different *Ipomoea* species and developmental stages.

Species	Eggs	Larvae	Prepupa	Pupa	Adults
*Ipomoea batatas*	82.61 ± 7.07 a	48.43 ± 9.88 a	60.72 ± 8.11 a	59.62 ± 12.69 a	59.84 ± 9.14 a
*Ipomoea hederifolia*	23.89 ± 6.98 d	43.31 ± 6.50 b	33.15 ± 6.01 b	28.87 ± 9.22 b	18.54 ± 9.53 b
*Ipomoea indica*	39.41 ± 5.67 b	20.34 ± 4.77 c	16.65 ± 3.59 c	16.53 ± 4.06 c	3.49 ± 3.99 c
*Ipomoea purpurea*	36.06 ± 7.17 c	18.24 ± 1.40 d	14.46 ± 4.00 d	14.62 ± 2.86 d	2.17 ± 0.51 d

Different letters within the same column indicate statistically significant differences according to Tukey’s test (*p* < 0.05).

## Data Availability

The raw data supporting the conclusions of this article will be made available by the authors on request.
